# Supplementary material for: Pharmacological Evidence Suggests That Slo3 Channel Is the Principal K+ Channel in Boar Spermatozoa
Source: Int J Mol Sci. 2023 Apr 25;24(9):7806. doi: 10.3390/ijms24097806 (PMC10178124; doi:10.3390/ijms24097806)
Supplement: Supplementary file 1 [file ijms-24-07806-s001.zip › ijms-2348385_supplementary titles.pdf]

Supplementary figures

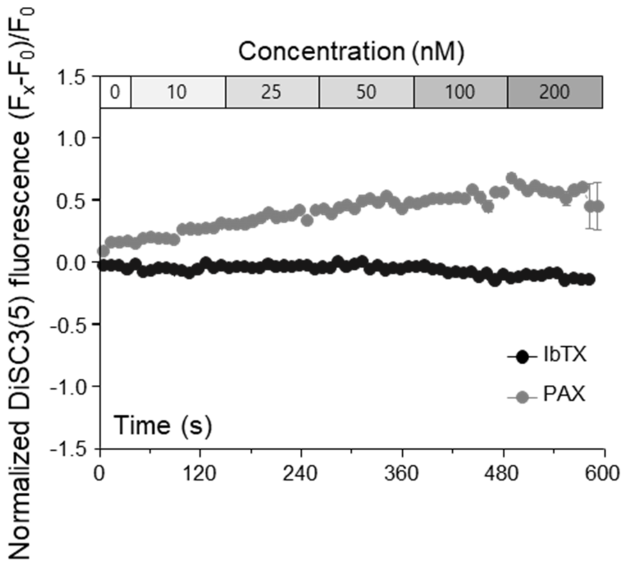

Supplementary Figure S1. Effect of specific Slo1 b lockers on boar sperm membrane potential

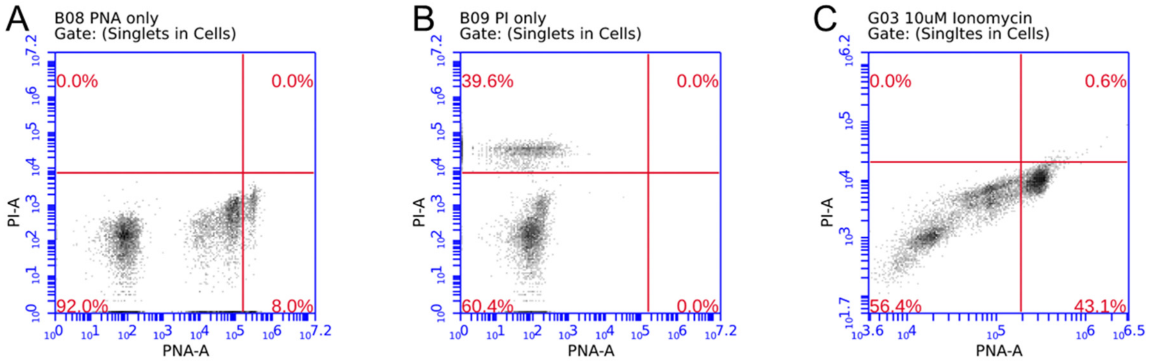

Supplementary Figure S2. PNA and PI single staining conditions and AR positive control

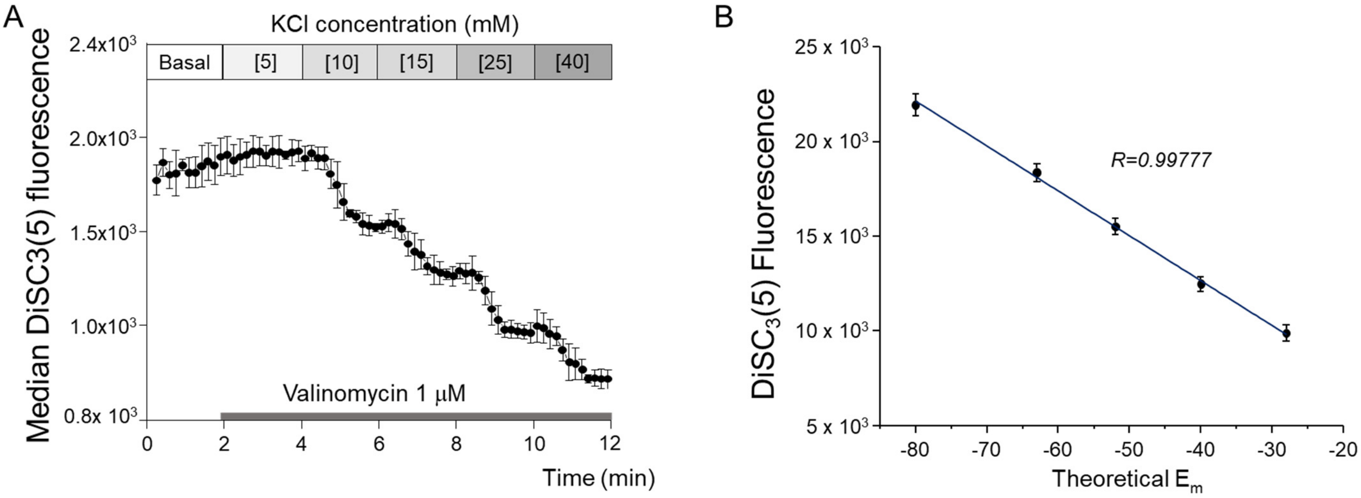

Supplementary Figure S3. Control experiments for DiSC3(5) fluorescence measurement of membrane potential.

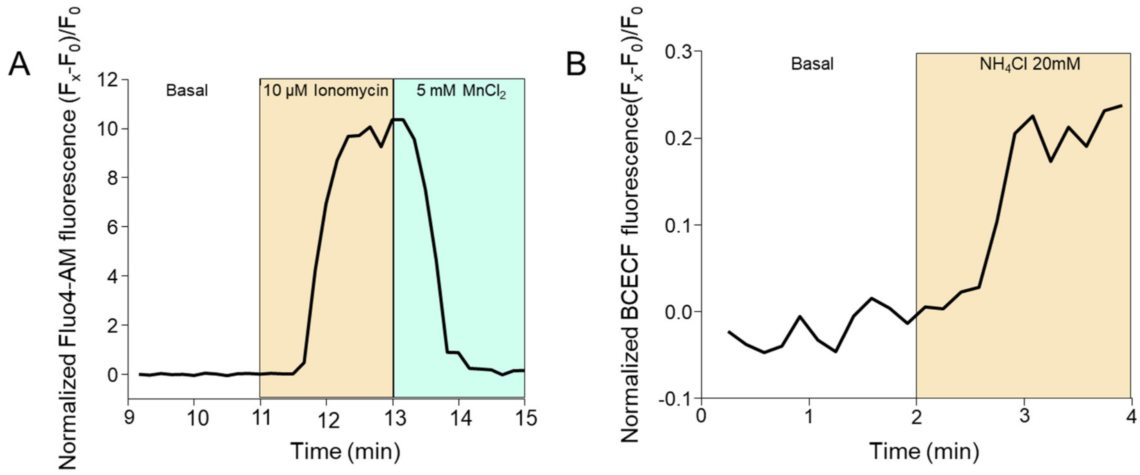

Supplementary Figure S4. Control experiments of fluorescence measurement for intracellular calcium (A, Fluoa-AM) and pH (B, BCECF).

Supplementary Table S1. Summary of the treatment concentrations

Technique	Parameter	Dye		Research question	Medium	Treatment	Treat-ment du-ration
**CASA**	**Motility**	NA	a)	Effects of LDD175 and NS1619 on motility and kinematic parameters	HTF	0, 1, 10, 100 µM LDD175;<br>0, 1, 10, 100 µM NS1619	5min
**TLFC**	**Em**	**DiSC3(5)** (25 nM)	a)	Em vs [K^+] calibration curve	NonCAP	▪ 0, 5, 10,15, 25, 40 mM KCl [1µM valinomycin was added at 2min time point]	2min for each treatment concentration
			b)	Em dose response to different K^+ channel modulators		▪ 0, 1, 10, 50, 100 µM LDD175<br>▪ 0, 1, 10, 50, 100 µM NS1619<br>▪ 0, 10, 25, 50, 100, 200 nM IbTx<br>▪ 0, 10, 25, 50, 100, 200 nM PAX	
			c)	Residual traces of K^+ current from Slo1		▪ 0, 10 µM LDD175, 100 nM IbTx<br>▪ 0, 100 nM IbTx, 10 µM LDD175<br>▪ 0, 10 µM LDD175, 100 nM PAX<br>▪ 0, 100 nM PAX, 10 µM LDD175	
	**$[Ca^{2+}]_i$**	**Fluo4-AM** (5µM), 0.02% Pluronic F-127	a)	Identification of [Ca^{2+}] responding subpopulation	NonCAP	▪ Basal, 10 µM Ionomycin, 5mM MnCl2	
			b)	Slo3 vs intracellular calcium signals		▪ 0, 1, 5, 10 µM LDD175<br>▪ 0, 1, 5, 10, 50, 100 µM NS1619	
			c)	Slo3 vs extracellular calcium independent [Ca^{2+}] signals	Ca-free medium	▪ 0, 1, 5, 10 µM LDD175<br>▪ 0, 1, 5, 10, 50, 100 µM NS1619	
			d)	Involvement of Slo3 in CatSper current modulation	NonCAP	▪ 0, 1µM NNC, 1, 5, 10 µM LDD175<br>▪ 0, 1µM NNC,5, 10, 50, 100 µM NS1619	

			e)	whether CatSper modulation is due to direct interactions with the chemicals of *Em* modulations		▪ 0, 20 mM TEA, 1, 5, 10 μM LDD175<br>▪ 0, 20 mM TEA,5, 10, 50, 100 μM NS1619	
	**$[pH]_i$**	**BCECF-AM** (300nM)	a)	Identification of pH responding subpopulation	NonCAP	▪ Basal, 20mM NH4Cl	
			b)	pH dose response to different K^+ channel modulators		▪ 0, 1, 10, 50, 100 μM LDD175<br>▪ 0, 1, 10, 50, 100 μM NS1619<br>▪ 0, 10, 25, 50, 100, 200 nM IbTx<br>▪ 0, 10, 25, 50, 100, 200 nM PAX	
**Flow cytometry**	**Acrosome reaction**	**PNA-FITC** (5ug/ml), **PI** (3μM)	a)	effect of LDD175 and NS1619 on AR	CAP	NC, 5 and 10 μM LDD175, 50 and 100 μM NS1619, PC (10 μM Ionomycin)	1hr

NA- not applicable, NC- negative control, PC- positive control, TLFC- time lapse flow cytometry
In TLFC, each bullet point (▪) represents a single treatment series and the treatments/concentrations of it are given in the order.
